# *Poria cocos* Solid-State Fermentation for Bioprocessing Tartary Buckwheat: Nutritional Enhancement, Antioxidant Activity, and Application in Steamed Bread

**DOI:** 10.3390/foods15101622

**Published:** 2026-05-07

**Authors:** Wendi Fan, Bowen Tan, Baokuo Song, Bin Zhang, Linlin Wang, Zhen Wang, Baojie Jiang

**Affiliations:** 1 College of science and Technology, Hebei Agricultural University, Cangzhou 061100, China; fwd81310424fwd@163.com (W.F.); tbw1910984321@163.com (B.T.); sbk15830703373@163.com (B.S.); zhmana@hotmail.com (B.Z.); lgwll@hebau.edu.cn (L.W.); wangzhen@hebau.edu.cn (Z.W.); 2 Hebei Key Laboratory of Analysis and Control of Zoonotic Pathogenic Microorganism, Baoding 071001, China

**Keywords:** *Poria cocos*, solid-state fermentation, Tartary buckwheat (TB), nutritional value

## Abstract

This study pioneered the application of *Poria cocos* solid-state fermentation (SSF) to Tartary buckwheat (TB). We aimed to enhance its nutritional value, antioxidant activity, and bioaccessibility through fungal biotransformation. Notably, SSF significantly increased protein (by 15.53%) and polysaccharides (by 158%) in TB, alongside a 4.87% improvement in DPPH radical scavenging capacity. Principal component analysis identified 5-day fermentation (FTB-5) as the optimal process, achieving the highest comprehensive score. Furthermore, SSF modified TB’s aroma profile (e.g., increasing 2(3H)-Furanone, 5-methyl by 352.02%) and optimized its amino acid composition, with essential amino acids rising by 12.90%. Critically, in vitro digestion confirmed a higher release rate of bioactive compounds in FTB-5 compared to raw TB. When applied to Chinese steamed bread, FTB-5 imparted a superior sensory score, with a soft texture and an appealing color. This study establishes *Poria cocos* SSF as a novel strategy for TB valorization, bridging traditional fungal biotechnology with functional food development.

## 1. Introduction

Tartary buckwheat (TB), a nutrient-rich cereal with demonstrated bioactivities such as antioxidation, anti-inflammation, anticancer, and antidiabetic effects, has gained increasing attention in functional food research [1]. These properties are attributed to its unique profile of bioactive compounds. For example, Huda et al. demonstrated that the flavonoids and phenolics present in buckwheat can be used in the treatment of various diseases, including glycemic disorders, diabetes, hypertension, microbial infections, cancer, and other related conditions [2]. However, the full potential of TB is limited by the poor bioaccessibility of its active components, primarily due to structural complexity and antinutritional factors (e.g., phytic acid and cellulose). To address this, strategies like germination, ultrafine grinding, and fermentation [3,4,5] have been explored to enhance nutrient release and transformation. Among these, solid-state fermentation (SSF) stands out for its ability to degrade macromolecules and antinutritional factors through enzymatic action, thereby improving grain digestibility and bioactive components content [6]. SSF has successfully enhanced bioactive components in substrates such as soybean and wheat bran using fungi like *Aspergillus* [7,8,9]. SSF with *Ganoderma lucidum* improves the nutritional quality and processing characteristics of wheat (*Triticum aestivum* L.) [10]. In addition, the obtained flour can be used to produce various food products, including meal replacement powder, bread [11,12,13], noodles [14], and so on.

Notably, among these fungi, *Poria cocos* (Schw.) Wolf (Polyporaceae), a medicinal-edible fungus, has shown unique advantages in fermenting traditional herbs and foods [15] and its long history of use also supports the safety of *Poria cocos* fermentation. First, as a brown-rot fungus, *Poria cocos* produces highly active cellulases and hemicellulases, with a dietary fiber degradation rate significantly higher than those of *Pleurotus tuber-regium* and *Polyporous rhinocerus* [16,17], though it lacks lignin-decomposing enzymes [18,19]. This trait prevents the degradation of lignin-like active components (tea polyphenols, flavonoids) during fermentation, thus retaining functional factors, while its cellulases convert cellulose and hemicellulose to notably boost polysaccharide content [20]. Second, studies indicated that the product fermented with *Poria cocos* has significantly higher polysaccharide and triterpenoid contents and stronger antioxidant activity compared to most edible fungi [20,21,22,23,24,25]. For example, Deng et al. reported that, compared with *Morchella* sp. and *Ganoderma lingzhi*, fermentation of pine pollen with *Wolfiporia cocos* not only exhibited the strongest ferric-ion reducing antioxidant power (FRAP) and a synchronous enhancement of multiple monomeric phenolic compounds but also maintained the greatest stability during the late fermentation stage [25]. Moreover, when fermenting with certain substrates, it generated unique volatile components, such as wild strawberry [26] and fruity aromas [20]. However, despite the proven potential of *Poria cocos* in enhancing bioactive components and antioxidant capacity via fermentation, no studies have yet explored *Poria cocos*-mediated SSF for TB modification, nor clarified how its metabolites synergize with TB components to amplify nutritional value—creating a critical research gap to address. We hypothesized that SSF with *Poria cocos* will significantly enhance the nutritional value, antioxidant activity, and bioaccessibility of TB by modifying its macromolecular structure and releasing bound bioactive compounds.

There have been reports on the fermentation of Tartary buckwheat using other microorganisms, such as *Monascus purpureus* [27] and amylolytic lactic acid bacteria [28]. However, studies on the fermentation of Tartary buckwheat using edible and medicinal fungi are still very limited. To improve the quality and increase the value of Tartary buckwheat, this study investigated the application of *Poria cocos* SSF to TB, systematically evaluating fermentation time effects on key nutritional markers (reducing sugars, proteins, polysaccharides), bioactive compounds (total phenolics, flavonoids, triterpenes), antioxidant activity (DPPH scavenging), nutrient release during in vitro digestion, and the sensory properties of TB-based Chinese steamed bread (CSB). Critically, principal component analysis (PCA) was employed to identify the optimal fermentation duration. We hypothesized that short-term SSF (5 days) would maximize bioaccessible compounds while longer fermentation would degrade them. To our knowledge, this is the first report integrating *Poria cocos* SSF with TB processing, bridging traditional medicinal wisdom with edible and medicinal applications. This finding not only advances SSF optimization strategies for underutilized grains but also provides a template for developing culturally relevant functional foods.

## 2. Materials and Methods

### 2.1. Materials

*Poria cocos*, provided by Hebei Agricultural University. TB, purchased from Chifeng, China. Amylase, pepsin and pancreatin, purchased from Beijing Merit Biotechnology Co., Ltd. (Beijing, China). All chemicals were of analytical grade.

### 2.2. Fermentation Process

TB SSF Medium: 40 g TB and distilled water were added into a conical flask (1:2.5, *w*/*v*). The TB sample was autoclaved at 121 °C and 15 psi for 30 min.

SSF process: Inoculate 1 cm^2^ of *Poria cocos* mycelium onto the TB SSF medium under sterile conditions. Once the mycelium had spread evenly across the surface, this point was designated as day 0. The samples were fermented at 28 °C for 5, 10, 15, 20, and 25 days, respectively (corresponding samples were labeled as FTB-5, FTB-10, FTB-15, FTB-20, and FTB-25). Each sample was prepared in three biological replicates and randomly placed in an incubator. To simultaneously terminate fermentation and dry the samples, the samples at different fermentation stages and the unfermented Tartary buckwheat (NFTB) were evenly spread out and placed in an oven at 55 °C for 24 h. They were then sieved through a 60-mesh sieve and stored at 4 °C.

### 2.3. Determination of Protein Contents

Nitrogen content in the samples was determined by the Micro-Kjeldahl method (GB5009.5-2016; National Food Safety Standard. Determination of Protein in Food. Beijing, China, 2016). The absorbance value was measured at 400 nm using an enzyme-linked reaction analyzer (FlexA-200, Hangzhou Allsheng Instruments Co., Ltd., Hangzhou, Zhejiang, China). The crude protein content was determined by multiplying the nitrogen content by a conversion factor of 6.25.

The protein nutritional quality was evaluated based on the amino acid profile according to the FAO 2013 adult reference pattern. The amino acid score (AAS) was calculated as the ratio of each essential amino acid content in the sample to the corresponding FAO reference value. The first limiting amino acid was identified as the essential amino acid with the lowest AAS. The protein digestibility-corrected amino acid score (PDCAAS) was calculated by multiplying the lowest AAS by the measured in vitro protein digestibility. The digestible indispensable amino acid score (DIAAS) was estimated using the total protein digestibility due to the lack of individual ileal amino acid digestibility data.

### 2.4. Determination of Total Triterpene Contents

Triterpenoid content was determined using the vanillin-acetic acid and perchloric acid colorimetric method [29] and was slightly modified. Then, the absorbance was measured at 550 nm with oleanolic acid as the reference standard. The standard curve is expressed by the equation: y = 305.59x − 12.391, where x is the absorbance and y is the total triterpene content. It should be noted that different triterpenoids may exhibit different colorimetric responses; therefore, the values represent relative total triterpene content rather than absolute concentrations of specific compounds.

### 2.5. Determination of Polysaccharide and Reducing Sugar Contents

The samples (1 g) were diluted with distilled water (99 g) and heated at 100 °C for 4 h, making the sample aliquot after centrifugation. The content of polysaccharides was determined using the phenol–sulfuric acid method. Briefly speaking, the sample aliquot was mixed with 5% phenol and sulfuric acid, and the absorbance was measured at 490 nm after standing for 30 min. A standard curve was established using glucose standards, yielding the equation: y = 0.0054x + 0.0385, where y is the absorbance, and x is the polysaccharide content. For the determination of reducing sugars, a sample aliquot was combined with a DNS reagent and subjected to boiling in a water bath for 5 min. After cooling, the absorbance was measured at 540 nm. A standard curve was established using glucose standards, yielding the equation: y = 0.483x − 0.021, where y is the absorbance and x is the reducing sugar content.

### 2.6. Determination of Flavonoid Contents and Total Phenol Contents

The samples (1 g) were diluted with an ethanol solution (80%, *v*/*v*) (24 g) and underwent ultrasound-assisted extraction for 2 h, making the sample aliquot after centrifugation at 6000 rpm for 15 min.

The flavonoid content was determined by the aluminum nitrate–potassium acetate colorimetric method. The sample solution (1.0 mL) was mixed with an equivalent amount of Al (NO_3_)_3_ (17.6 g of AlH_6_NO_4_ was dissolved in water and diluted to a final volume of 100 mL) and CH_3_COOK (9.814 g of CH_3_COOK was dissolved in water and diluted to a final volume of 100 mL). The absorbance was measured at 420 nm after standing for 1 h. Rutin is used as the standard. The standard curve is expressed by the equation: y = 0.1351x + 0.0451, where y is the absorbance and x is the flavonoid content.

The total phenolic content was determined using the Folin–Ciocalteu method. Briefly speaking, the sample solution (1.0 mL) was mixed with 1.0 mL of Folin–Phenol (BR, 1 mol/L) and 10.6% sodium carbonate (4.0 mL) incubated for 1 h. The absorbance was measured at 760 nm. Gallic acid is used as the standard. The standard curve is expressed by the equation: y = 0.0875x + 0.0574, where y is the absorbance and x is the total phenolic content.

### 2.7. Determination of DPPH Free-Radical Scavenging Activity

The sample solution was prepared, refer to Section 2.6. It (1.0 mL) was mixed with a DPPH solution (3.0 mL), designated as As. The sample solution was mixed with ethanol, designated as Ac. The DPPH solution was mixed with ethanol, designated as Ab.

All mixtures were incubated in the dark at room temperature for 30 min. The absorbance was measured at 517 nm. The DPPH radical scavenging capacity was calculated using the following formula:

The DPPH radical scavenging capacity=(1−(As−Ac)/Ab)×100%

### 2.8. Determination of Phytic Acid, Tannin Content, and α-Amylase Inhibitory Activity

The phytic acid and tannin content assays were performed using commercial kits from BIOESN (Shanghai, China). The catalog numbers are provided below for reproducibility: Phytic acid assay kit: Catalog No. BES-BK20; and Tannin content assay kit: Catalog No. BES-BK2699B.

α-Amylase inhibitory activity was measured as described by Zhang et al. [30]. Briefly, 100 μL of the sample solution was mixed with 200 μL of the α-amylase solution and 100 μL of a 2 mM phosphate buffer and incubated for 20 min. Subsequently, 100 μL of a 1% starch solution was added. In the control, the enzyme solution was replaced by an equivalent volume of buffer. After 5 min, 500 μL of 3,5-dinitrosalicylic acid (DNS) reagent was added to terminate the reaction, and the samples were heated in a boiling water bath for 5 min. Absorbance was measured at 540 nm and the α-amylase inhibition rate was calculated using the following formula: Inhibition rate = (1 − (A − A_0_)/(A_1_ − A_0_)) × 100%.

Wherein A is the absorbance of the sample solution, A_0_ is the absorbance of the blank control, and A_1_ represents the absorbance of the control group solution.

### 2.9. Determination of Amino Acid Composition

To determine the amino acid composition and content in the samples, a combination of acid hydrolysis and high-performance liquid chromatography (HPLC) was employed. Briefly, 0.2 g of the sample was mixed with 10 mL of 6 mol/L hydrochloric acid containing 0.1% phenol and placed in a 35 mL acid hydrolysis tube. The mixture was then hydrolyzed at 110 °C for 22 h. Analysis was conducted using a Waters Symmetry C18 column (4.6 × 250 mm, 4.6 μm). The HPLC conditions were as follows: column temperature, 40 °C; detection wavelength, 360 nm; injection volume, 10 μL; flow rate, 1.5 mL/min; and run time, 30 min. The mobile phase consisted of two solvents: A, acetonitrile; B, an acetic acid-sodium acetate buffer. The gradient elution was set as follows: 18% A from 0 to 6.5 min, 18 to 20% A from 6.5 to 10 min, 20–34% A from 10 to 20 min, 34–45% A from 20 to 23 min, 45–55% A from 23 to 25 min, 55–18% A from 25 to 27 min, and finally 18% A from 27 to 30 min. The content of each amino acid in the sample was calculated using the single-point external standard method, following the formula below: X_i_ = A1/A0 × C_0_ × K/m × 100/1000. Wherein, X_i_: the content of specific components in the sample, %;A0: the peak area of specific components in the standard solution; C_0_: the concentration of specific components in the standard solution, mg/mL; A1: the peak area of each specific component in the sample solution; K: the volume of the sample after constant volume, mL; m: the weight of the sample; and g: 100/1000—unit conversion.

### 2.10. Determination of Volatile Components

Volatile components were analyzed by GC-IMS (Shandong Hanon Scientific Instruments Co., Ltd., Dezhou, China) using the following parameters: The GC column used was an MXT-WAX capillary column (15 m × 0.53 mm, 1.0 μm). High-purity nitrogen (≥99.999%) was used as both the carrier gas and the drift gas, with a drift gas flow rate of 150.0 mL/min. The mixed standard contained 2-butanone, 2-pentanone, 2-hexanone, 2-heptanone, 2-octanone, and 2-nonanone (all ≥99% purity). This instrument’s LOD for volatile compounds is at the ppb level, which is suitable for trace VOC analysis. The operation steps are as follows: each sample (3 g) was transferred into a 20 mL vial, incubated at 80 °C for 15 min, and then analyzed. A total of 200 μL was injected from the headspace using a heated syringe (at 110 °C). The carrier gas was N_2_ with a purity of 99.999%. The programmed flow rates and durations were as follows: 2 mL/minutes for 2 min, 10 mL/minutes for 8 min, and 100 mL/minutes for 20 min. The analytes were pushed into the ionization chamber and then sent to the IMS detector. The ionization source of the IMS was tritium. A mixed standard of 6 ketones was analyzed to establish calibration curves for the retention time and retention index. Subsequently, the retention index of the target analyte was calculated from its retention time. The obtained spectra were qualitatively and quantitatively analyzed by using the GC-IMS library in VOCal.

### 2.11. Methods for Identification of Key Volatile Components

According to the suggestion of Pei [31], the relative odor activity value (ROAV) method combined with the variable importance in projection (VIP) value was adopted to analyze the key flavor compounds during the fermentation process of TB. The formula for calculating ROAV is as follows: In this formula, CA represents the relative percentage content of compound A. TA represents the aroma threshold of compound A, which can be obtained from the literature in units of mg/kg [32,33]. Tstan refers to the aroma threshold of the compound that contributes most significantly to the sample flavor, while Cstan refers to its relative percentage content. ROAV = (CA/TA) × (Tstan/Cstan) × 100. The odor information of aroma compounds comes from the scientific literature (Compilations of odor threshold values in air, water and other media; characteristic aroma components in tobacco extracts from You Xi based on odor activity value) and the Flavornet database. (http://www.flavornet.org. accessed on 21 April 2025).

### 2.12. In Vitro Digestion Simulation

Simulated oral digestion phase: The sample (2.0 g) was mixed with physiological saline (4.0 mL, 0.9%, *w*/*v*) and a saliva amylase solution (1.0 mL, 750 U/mL). The mixture was incubated for 5 min at 37 °C.

Simulated Gastric Digestion Phase: The sample from the oral digestion phase was mixed with HCl (9.0 mL, 0.02 M). The mixture was adjusted to pH 2.0 using HCl (1 M), and then a pepsin solution (1.0 mL, 2000 U/mL) was added. The mixture was incubated for 2 h at 37 °C, with sampling conducted every 30 min.

Simulated intestinal digestion phase: The gastric digestion sample was mixed with a phosphate buffer (9.0 mL, pH 6.8), and that was adjusted to pH 6.8 using NaOH (1 M). Then, the mixture was mixed with a trypsin solution (1.0 mL, 800 U/mL). The mixture was incubated for 2 h at 37 °C, with sampling conducted every 30 min.

Post-digestion phase: After each digestion phase, the samples were placed in a water bath at 70°C for 3 min to terminate the enzymatic process. The samples were centrifuged at 6000 rpm for 15 min, and a clear supernatant was obtained and stored at −20 °C for further analysis.

### 2.13. Scanning Electron Microscope Tests

Scanning electron microscope (SEM) tests were conducted to compare the SEM images of NTB and FTB structures. A small amount of sample was directly mounted onto a conductive adhesive, and sputter-coated with gold for 45 s at 10 mA using a Quorum SC7620 sputter coater (Quorum Technologies Ltd, Lewes, UK)). The morphology of the samples was subsequently observed using a TESCAN MIRA LMS SEM.

### 2.14. Preparation of TB CSB

TB CSB was designed with reference to Xu’s work and slightly modified according to actual conditions [34]. TB CSB were prepared by the following method. Firstly, 460 g of wheat flour, 2.5 g of Angel high-activity yeast, 40 g dried NTB/FTB flour, and an appropriate amount of warm water were kneaded into a dough with a uniform and soft texture and a smooth surface. Then, these parts were transferred to a constant temperature water bath, sealed, and fermented at 35 °C for 60 min. Subsequently, 2 g of baking soda was added and kneaded evenly to remove the air inside. The dough was divided into pieces of about 50 g each, shaped into hemispheres, and proofed for 10 min. Then, the dough was steamed in boiling water for 25 min and then cooled at room temperature for 30 min to obtain CSB samples [35].

### 2.15. Determination of the Color and Specific Volume of CSB

The color was measured using a portable colorimeter colorimeter (Model SC-10, Shenzhen 3nh Technology Co., Ltd., Shenzhen, China). In the data, L* represents brightness (ranging from black [0] to white [100]), a* represents the range from green (−) to red (+), and b* represents the range from blue (−) to yellow (+). The ΔE of FTB was calculated using NFTB as the reference, and the ΔE of CSB was calculated relative to the CSB containing NFTB. The specific volume was determined using the millet displacement method. The calculation formula of ΔE was as follows: ∆E = $\sqrt{\Delta L^{2}+\Delta a^{2}+\Delta b^{2}}$.

### 2.16. Determination of Taste Characteristics of CSB Based on Electronic Tongue

The samples were ground to 40 mesh, sealed, and stored in a dry environment until analysis. Before testing, 1.0 g of each sample was accurately weighed into a 100 mL volumetric flask, diluted to volume with distilled water, and extracted ultrasonically in a 50 °C water bath for 30 min. The extract was centrifuged at 8000 rpm for 10 min and filtered through a 0.45 μm microporous membrane. The supernatant was stored at 4 °C until analysis.

Taste analysis was performed using an INSENT SA402B taste sensing system (INSENT Inc., Atsugi, Japan), which can objectively and digitally evaluate sourness, bitterness, astringency, aftertaste-B, aftertaste-A, umami, richness, saltiness, and sweetness.

Before measurement, the sensors were cleaned sequentially with cleaning solution 1 (90 s), cleaning solution 2 (120 s), and cleaning solution 3 (120 s) to ensure cleanliness. The sensors were then conditioned in a reference solution (30 mM KCl + 0.3 mM tartaric acid) for 30 s, repeated 20 times, to achieve stabilization and baseline calibration. The negative cleaning solution was a mixture of 30% (*v*/*v*) ethanol and 100 mM HCl, and the positive cleaning solution was a mixture of 30% (*v*/*v*) ethanol, 100 mM KCl, and 10 mM KOH.

During sample measurement, an appropriate amount of sample solution was placed in a sample cup, and the sensor was immersed for 30 s to collect signals. The system automatically recorded the response values. After each measurement, the sensor was cleaned in cleaning solutions 4, 5, and 6 (3 s each) to remove residual components, followed by 3 s in a reference solution to return to baseline, and then equilibrated in a CPA solution for 30 s before the next measurement. Taste intensity was expressed as relative potential.

### 2.17. Determination of Aroma Characteristics of CSB Based on Electronic Nose

An accurate 2 g sample was placed in a 20 mL headspace vial, sealed, incubated at 40 °C for 5 min, and then analyzed. Volatile fingerprint analysis was performed using a PEN3 portable electronic nose system (Airsense Analytics GmbH, Schwerin, Germany), equipped with 10 metal oxide sensors showing selective responses to different volatile compounds: W1C (aromatics), W5S (nitrogen oxides), W3C (ammonia/aromatics), W6S (hydrogen), W5C (alkanes/aromatics), W1S (methane), W1W (sulfides/organics), W2S (alcohols), W2W (aromatics/sulfur compounds), and W3S (alkanes/methane).

The parameters were set as follows: sample preparation time: 5 s, data acquisition time: 120 s, sensor cleaning time: 60 s, injection flow rate: 400 mL/min, and filtered clean air was the carrier gas. A randomized block design was applied with three replicates (1 g per replicate). All analyses were performed at 25 ± 1 °C and 50 ± 5% relative humidity.

Time-series data of sensor responses (G/G_0_, where G is the conductance during sample measurement and G_0_ is the baseline in clean air) were recorded. Steady-state response values were extracted as characteristic parameters. Data from sensors W1C, W3C, and W5C were inverted before analysis, while data from the other sensors were used directly.

Sample preparation was consistent with Section 2.16 (electronic tongue). Briefly, 3.0 g of liquid sample was transferred into a 10 mL headspace vial, kept at 25 °C for 30 min, and then analyzed by headspace injection using the same 10-sensor electronic nose system. The flow rate was 400 mL/min, the sample preparation time was 5 s, the data acquisition time was 120 s, and the sensor cleaning time was 60 s. The operating conditions were 25 ± 1 °C and 50 ± 5% relative humidity.

### 2.18. Sensory Evaluation of CSB

A trained sensory panel consisting of 10 postgraduate students (5 males and 5 females, aged between 20 and 35 years old) majoring in Biology and Medicine from Hebei Agricultural University conducted sensory evaluations on the CSB samples.

### 2.19. Statistical Analysis

All experiments were independently repeated three times. The data were expressed as mean ± standard deviation (SD). Normality of the data was assessed using the Shapiro–Wilk test. One-way analysis of variance (ANOVA) followed by Tukey’s post-hoc test was used for multiple comparisons, with statistical significance set at *p* < 0.05. PCA was performed using SPSS 26.0, and orthogonal partial least squares discriminant analysis (OPLS-DA) was conducted using SIMCA 14.1. Graphs were generated using Excel 2019.

## 3. Results and Discussion

### 3.1. The Effect of Fermentation on Nutrients, Antioxidant Activity, and PCA-Based Screening

SSF is an effective way to enhance the active compounds and antioxidant capacity of grains [6]. The dynamic changes of various nutrients and antioxidant activities during the fermentation process are shown in Table 1 below. During the fermentation process, the protein content of the fermented sample showed a trend of first increasing and then decreasing, reaching the highest value by the 5th day (119.00 ± 0.82 mg/g). This indicated that long-term fermentation of *Poria cocos* is not conducive to the accumulation of protein. This was consistent with the results reported by Heidari et al. [36]. The moderate increase in protein (+15.53%) likely results from both de novo microbial protein synthesis and a concentration effect due to the degradation of non-protein dry matter. The change trends of the contents of polysaccharides, reducing sugars, and triterpenes were similar: showing a fluctuating situation of first increasing, then decreasing, then increasing again, and finally decreasing. Among them, the contents of both polysaccharides and reducing sugars reached their highest values on the 15th day, which were 669.29 ± 6.45 mg/g and 549.30 ± 8.98 mg/g respectively, while the content of triterpenes reached its highest value of 14.02 ± 0.94 mg/g on the 20th day. The substantial increase in FTB-5 polysaccharides (+158%) is primarily attributed to the biosynthesis of *Poria cocos*-specific polysaccharides (e.g., β-glucans) during fermentation, as supported by the concurrent rise in reducing sugars. The content of flavonoids significantly increased and reached 31.73 ± 1.18 mg/g on the 20th day. The content of total phenolics reached its highest value of 18.83 ± 0.05 mg/g on the 15th day. Flavonoids were the main constituent of phenols, and TB contains high levels of flavonoids, contributing to its high antioxidant activity [12]. Recent studies have shown the ability of SSF of fungi to release phenolic compounds and enhance biological activities [6]. The DPPH free radical scavenging activity showed a trend of first increasing and then decreasing. It reached the highest value of 95.83 ± 1.14% on the 5th day and then gradually decreased afterwards. The change in DPPH free radical scavenging activity was related to the changes in the contents of various active components. In the early stage of fermentation, the combined effects of factors such as the increase in the contents of antioxidant-related components (such as flavonoids, phenols, polysaccharides) led to the improvement of free radical scavenging activity [37]. In the later stage, with the changes in the contents of these components or the variations in their interactions, the scavenging activity decreases. The research results indicated that SSF of *Poria cocos* enhanced the antioxidant capacity of TB by increasing the release of active substances. This further demonstrates the potential of edible fungus mycelium fermentation for improving the functional properties of Tartary buckwheat in food applications. This was consistent with the results reported by Zhou et al. Their research results showed that the fermentation of six medicinal fungi including *Ganoderma lucidum* increased the content of active ingredients and antioxidant activity in barley, further demonstrating that SSF by fungi can improved the nutritional and functional values of cereals [38].

PCA was utilized to obtain principal component factors that retained more than 90% of the differences in the original data. Then, combined with the percentage of their explanatory variables, a comprehensive score was calculated and used to evaluate the quality of TB fermented by *Poria cocos*. PCA was one of the most frequently used cluster display analysis methods in the field of food chemistry. It performed dimensionality reduction on multiple correlated factors to obtain a few principal component factors that are uncorrelated with each other while retaining as much of the differences from the original data as possible. Using a few principal component factors could explain the variations in a large amount of original data [39,40]. Using PCA to describe the quality indicators of TB fermented by *Poria cocos* was more accurate than using a single indicator, and at the same time, it avoided the influence of multiple correlated indicators on the evaluation results [41]. In this study, as can be seen from Table 2, the eigenvalues of the first three factors are 3.214, 2.225, and 1.374 respectively. Their variance contribution rates were 45.909%, 31.787%, and 19.634% respectively, and the cumulative variance contribution rate reached 97.331%, indicating that the information contained in the selected three principal component factors exceeds 90% of the information of the original variables. Through comprehensive analysis, selecting three principal component factors simplifies the number of original variables while containing as much information of the original variables as possible. Therefore, it was quite appropriate to choose three principal component factors. The PCA loading matrix represented the correlation coefficients between the principal component factors and the original variables [42]. Generally, 0.5 was taken as the basis for judging the correlation [42]. As shown in Table 3, PC1 explained 45.909% of the total variance and was mainly driven by changes in reducing sugar, polysaccharide, and total phenolic contents, reflecting the fermentation-induced accumulation of primary metabolites and phenolic compounds. PC2 explained 31.787% of the variance and was associated with triterpenes and flavonoids, suggesting a distinct biochemical trajectory related to the enhancement of lipophilic bioactive components. PC3 explained 19.634% of the variance and was linked to protein content and the DPPH free radical scavenging rate, indicating a correlation between protein alteration and antioxidant capacity during fermentation. Based on the scores of each principal component (Y1–Y3), taking the proportion of the eigenvalues corresponding to the three principal components to the sum of the eigenvalues of all the extracted principal components as the weight, a comprehensive evaluation model was constructed as follows [43]: Y = ((3.214/(3.214 + 2.225 + 1.374 = 6.813)) × Y1 + (2.225/6.813) × Y2 + (1.374/6.813) × Y3). According to this model, the comprehensive principal component values of each group were calculated, as shown in Table 4. The top one, in terms of the comprehensive total scores, was FTB-5. It can be intuitively observed that FTB-5 obtained the highest score in comprehensive quality, which indicates that its overall performance in various evaluated indicators is superior to other groups. Therefore, in subsequent research, we will focus on analyzing the differences between FTB-5 and NTB.

### 3.2. The Effect of Fermentation on Functional Activity and Nutritional Composition

The nutritional value of proteins in grains can be related to their ability to meet nitrogen and essential amino acid requirements [44,45]. Table 5 shows the amino acid composition and content of TB before and after fermentation. It was worth noting that threonine, valine, isoleucine, leucine, and phenylalanine are essential amino acids for the human body [46], all of which were elevated in FTB-5. Threonine and valine increased to 26.31% and 11.73%, respectively. Moreover, aspartic and glutamic contributed to the sweetness and freshness of TB [47]. Methionine had the function of sustaining and regulating growth and development [48]. The content of these amino acids has been increased during fermentation, in this study. Furthermore, the lysine/arginine and methionine/glycine ratios were reduced in FTB-5. Emerging scientific evidence has established that the molar ratios of lysine-to-arginine (Lys/Arg) and methionine-to-glycine (Met/Gly) serve as critical determinants modulating the hypocholesterolemic efficacy of plant-derived proteins. Current research demonstrates an inverse relationship between these amino acid ratios and cholesterol-lowering capacity, with diminished ratios correlating with enhanced therapeutic outcomes [11]. Based on essential amino acid composition and protein digestibility, the PDCAAS and estimated DIAAS values were calculated. For NTB, the first limiting amino acid was methionine + cysteine with an AAS of 0.636. The protein digestibility was 70.10%, leading to a PDCAAS of 0.446 and an estimated DIAAS of 0.446. For FTB-5, the first limiting amino acid was also methionine + cysteine with an AAS of 0.453. The protein digestibility was 75.69%, resulting in a PDCAAS of 0.343 and an estimated DIAAS of 0.343. Both samples showed moderate-to-low protein nutritional quality, which is typical for cereal bran proteins.

Table 6 shows the antioxidant activity, antinutritional factors (phytic acid and tannin), and α-amylase inhibitory activity of TB before and after fermentation. The antioxidant activity of TB exhibited a differential response to *Poria cocos* fermentation, depending on the specific assay used. The ABTS radical scavenging activity significantly decreased from 71.07% to 40.17%. In contrast, the DPPH radical scavenging activity and Fe^2+^ chelating capacity significantly increased from 90.96% to 95.83% and from 14.11 to 16.39 μmol/g, respectively. This discrepancy suggests that fermentation selectively modulates the antioxidant profile of TB. The decrease in ABTS activity may be attributed to the microbial consumption of certain labile phenolic compounds, while the increase in DPPH and Fe^2+^ chelating activities indicates the generation or enhanced extractability of other potent antioxidants, such as small peptides, Maillard reaction products, or specific phenolic derivatives. Regarding antinutritional factors, the phytic acid content significantly decreased from 2.99 to 2.60 mg/g. This reduction is a well-documented beneficial effect of fermentation, primarily caused by microbial phytases that hydrolyze phytic acid, thereby improving mineral bioaccessibility. Conversely, tannin content showed no significant change (*p* > 0.05), indicating stability under the applied fermentation conditions. Notably, α-amylase inhibitory activity significantly increased from 53.94% to 71.87% (*p* < 0.05). This enhancement suggests the formation or release of potent inhibitors, such as polyphenols or bioactive peptides, which could delay starch digestion and reduce postprandial blood glucose, offering anti-hyperglycemic benefits. Collectively, these changes provide a robust scientific basis for selecting this fermented product as a functional food ingredient.

### 3.3. The Effect of Fermentation on Aroma Profile

**Figure 1:**
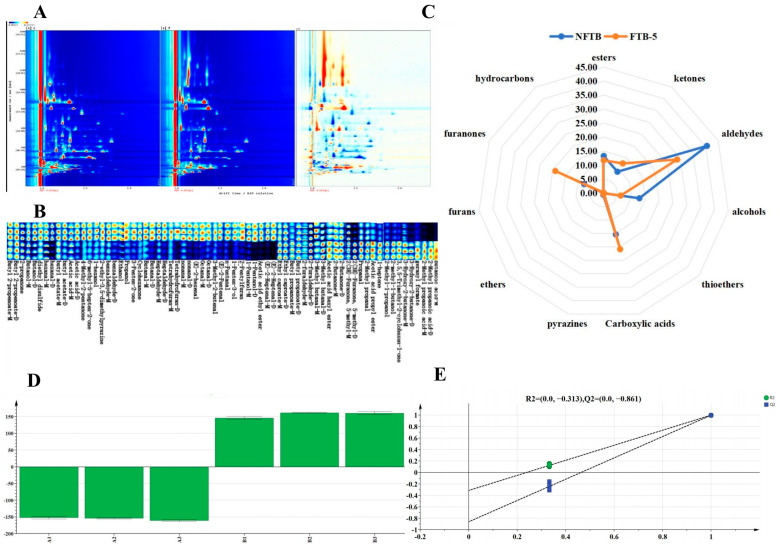
The two-dimensional chromatograms of GC-IMS (**A**). Fingerprint spectra in TB before and after fermentation (**B**). Radar chart of relative content of volatile components in TB before and after fermentation (**C**). OPLS-DA (**D**). Permutation test of permutation test of OPLS-DA model (**E**).

Signature volatile organic compounds (VOCs) play a pivotal role in maintaining organoleptic consistency and the flavor profile stability of artisanal fermented products. These biochemical markers serve as critical parameters for monitoring microbial metabolic activities and regulating bioprocess dynamics during fermentation [49,50]. Therefore, this study conducted an analysis of the aroma profile of TB before and after fermentation. GC-IMS was employed to examine the volatile components in TB before and after fermentation. The two-dimensional chromatograms of GC-IMS are illustrated in Figure 1A, in which the morphological images of NFTB were selected as references. The horizontal axis denoted the ion mobility time, while the vertical axis represented the retention time of gas chromatography. The red vertical line signified the reaction ion peak (RIP), and the signal intensity was depicted by the color [51]. Figure 1A distinctly reveals notable discrepancies between the two chromatograms, with more red areas evident in the comparison chart. This indicated the contents of certain substances in TB that after being fermented by *Poria cocos* have increased, showing remarkable effects. Complementary to the multivariate analysis, comparative volatile profiling was conducted through chromatographic fingerprinting analysis, as illustrated in Figure 1B. This two-dimensional visualization revealed significant metabolic shifts in TB volatilomes before and after fermentation, with aldehydes and alcohols demonstrating marked attenuation in chromatographic signal intensities. These observations exhibited strong concordance with the quantitative compositional changes presented in Figure 1C.

GC-IMS detected 48 conserved volatile organic compounds across both fermentation states, classified into 11 functional groups: 15 aldehydes, 8 alcohols, 8 esters, 8 ketones, 3 carboxylic acids, 1hydrocarbons, 1ethers, 1furans, 1 thioethers, 1 furanones, and 1pyrazines. Notably, while compound diversity remained invariant, significant quantitative redistributions were observed as shown in Figure 1C. Overall, after fermentation, the relative contents of alcohols, furans, and ethers in the TB decreased significantly. Additionally, the relative contents of esters, thioethers, pyrazines, and aldehydes decreased slightly, while the relative contents of furanones and hydrocarbons increased significantly. Moreover, the relative contents of ketones and carboxylic acids increased slightly. The changes can be attributed to the metabolic activities of *Poria cocos*. The decrease in alcohols likely results from their oxidation to aldehydes and subsequent conversion to carboxylic acids via alcohol dehydrogenase and aldehyde dehydrogenase pathways, which is consistent with the observed slight increase in carboxylic acids. The reduction in furans and ethers may reflect their utilization as carbon sources or their degradation by fungal extracellular enzymes such as laccases and peroxidases. In contrast, the marked increase in furanones and hydrocarbons suggests the generation of Maillard reaction intermediates and lipid-derived volatiles, which are often enhanced during fungal fermentation [52]. The slight changes in esters and thioethers indicate that their formation and degradation reached a near equilibrium under the current fermentation conditions. TB naturally contains a variety of aldehydes, and these aldehydes were part of the volatile components of TB [53]. With their low sensory thresholds, aldehydes played an important role in the formation of the flavor of TB, bringing unique aroma and flavor characteristics to it [54].

As can be seen from Table 7, among the 24 compounds, the main aldehydes are benzaldehyde (D, M), heptaldehyde (D, M), (E)-2-heptenal (D, M), butanal (D, M), Octanal (D, M), nonanal (D, M), 3-methyl butyraldehyde (D, M), hexanal (D, M), and 2-furaldehyde (D, M). Among them, hexanal (D, M) is regarded as an important volatile flavor component, which could bring grassy and tallowy notes to TB. With the exception of propanal, D-3-methyl butyraldehyde, and D-2-furaldehyde, the relative concentrations of the remaining aldehyde compounds all decreased to varying degrees after fermentation, resulting in an overall decrease in the relative content of aldehydes. Alcohols and carboxylic acids had a relatively high odor threshold and usually do not have a significant impact on the flavor of TB [55]. Since esters were not important volatile flavor components in TB in this study, no detailed analysis will be carried out here. Although the overall content of ketones increased, the contents of all except 3-Hydroxy-2-butanone (D, M) and 3,5,5-Trimethyl-2-cyclohexen-1-one decreased. 3-Hydroxy-2-butanone (D, M) was an important volatile flavor component in FTB, which can bring a creamy aroma to TB. In addition, furans, ethers, pyrazines, thioethers, furanones, and hydrocarbons were also found in the TB. After fermentation with *Poria cocos*, the relative concentrations of furanones and hydrocarbons increased, while those of the other several types decreased. Furanones were characterized by a low odor threshold, which meant they can have a significant impact even at low concentrations, endowing TB with a spicy, roasted sweet aroma [56].

OPLS-DA was employed to effectively discriminate between pre-fermentation and post-fermentation datasets under supervised conditions, enabling the precise construction of inter-group differential metabolic profiles [57]. As illustrated in Figure 1D, the model demonstrated significant metabolic divergence in VOCs between the non-fermented and fermented samples, with exceptional intra-group reproducibility observed. The established model exhibited robust performance metrics: the explained variance for descriptor variables (R^2^X) reached 0.974, while the dependent variable explanation (R^2^Y) attained 0.999. Corresponding predictive accuracies on the X- and Y-axes were quantified as 97.4% and 99.99%, respectively. Notably, the model’s predictive capability (Q^2^) achieved 0.998, corresponding to a recognition rate of 99.8%. These parameters substantially exceed the minimum acceptance threshold of 0.50 for both R^2^ and Q^2^ values, with proximity to unity indicating optimal model performance [58]. Validation through 200 permutation tests (Figure 1E) confirmed model robustness, as evidenced by the Q^2^ regression line intersecting the vertical axis at a negative value, thereby excluding overfitting concerns [59]. The very high R^2^Y and Q^2^ values should be interpreted with caution, as they may suggest overfitting given the limited sample size. Permutation testing (*p* < 0.05) supported model validity, but we recognize that additional validation (e.g., external test set) would be required to confirm robustness. We have clarified that no external validation set was used, which is a limitation.

Variable Importance in Projection (VIP) analysis identified 17 discriminant metabolites (VIP > 1.0) that significantly influenced volatile profile alterations in Tibetan barley (TB) during fermentation (Table 8). A subsequent flavor impact assessment employed a relative odor activity value (ROAV) methodology, where compounds with ROAV ≥ 1.0 were designated as key aroma-active constituents [60]. This analysis revealed 12 critical flavor compounds, including (E)-2-Heptenal-M, 2-furaldehyde-D, hexanal-D, and hexanal-M, characterized by elevated ROAVs. These components conferred complex aromatic attributes encompassing grassy, fruity, tallowy, burnt, floral, and almond oil notes to TB matrices. Notably, the post-fermentation samples exhibited significant ROAV enhancement for 11 of 12 key volatiles. Particularly pronounced increases were observed in 2-Methyl propanoic acid-M (isobutyric acid), 3-Hydroxy-2-butanone-D (acetoin), 2-Methyl propanoic acid-D, and butanoic acid-M, compounds associated with desirable dairy-associated flavor profiles including butter, cheese, and cream notes. This metabolic shift may suggest that SSF could effectively enrich TB with favorable volatile constituents, which might modulate its organoleptic properties via the activation of related biosynthetic pathways. The observed VOC profile modification suggests targeted microbial activity during fermentation drives the biosynthesis of sensory value metabolites, ultimately enhancing product aroma complexity.

### 3.4. The Effect of Fermentation on Various Nutrients, Antioxidant Activities, and Microstructure During the In Vitro Digestion Process 

**Figure 2:**
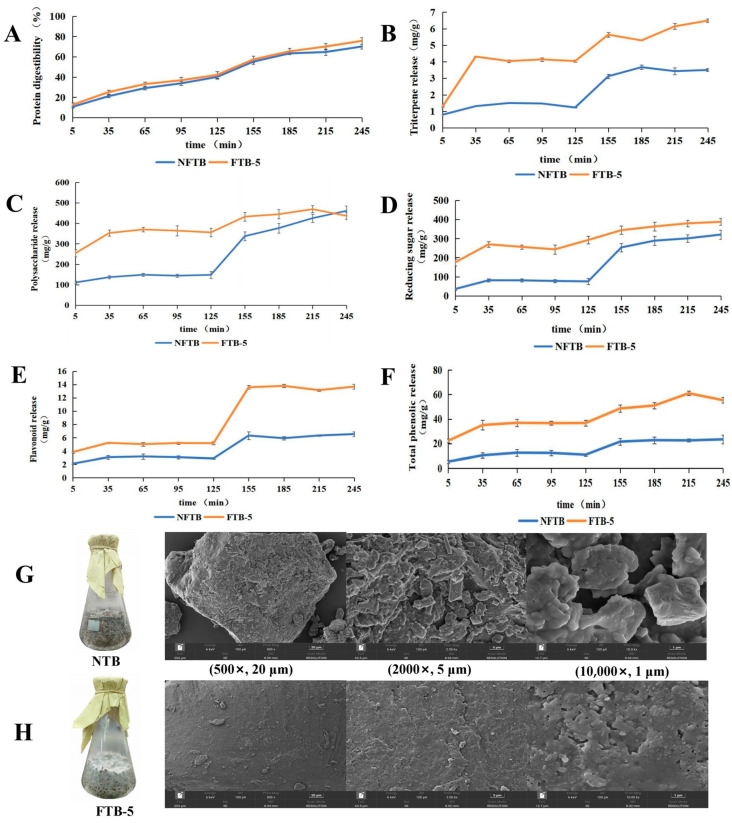
Release kinetics of simulated Nutritional substances’ in vitro digestion of fermented products of TB at different periods: (**A**) protein, (**B**) triterpenoids, (**C**) polysaccharides, (**D**) reducing sugars, (**E**) flavonoids, and (**F**) total phenolic. Macroscopic and electron microscope images of TB before (**G**) and after fermentation (**H**).

The release kinetics of simulated Nutritional substances’ in vitro digestion of fermented products of TB at different periods are shown in Figure 2A–F. Throughout the digestion process and over the entire time course, the protein digestibility and the release amounts of polysaccharides, reducing sugars, triterpenes, total phenols, and flavonoids of FTB-5 were always higher than those of NFTB. Initially, the release amounts of bioactive substances of both were low. As time increased, the protein digestibility and the release rates of bioactive substances of both gradually rose, but the rising speed of FTB-5 was faster. At 245 min, protein digestibility reached 75.69 ± 3.36%. The release amounts of reducing sugars and triterpenes were also the highest, reaching 387.80 ± 18.07 mg/g and 6.49 ± 0.11 mg/g, respectively. At 215 min, the release amounts of polysaccharides and total phenols were the highest, reaching 468.99 ± 17.14 mg/g and 60.99 ± 1.80 mg/g, respectively. At 185 min, the release amounts of flavonoids were the highest, reaching 13.78 ± 0.26 mg/g. These six substances (graphs A–F) collectively showed that during in vitro digestion, FTB-5 was significantly superior to NFTB in terms of protein digestibility, polysaccharide release amount, reducing sugar release amount, triterpene release amount, total phenol release amount, and flavonoid release amount. These results further illustrated that the fermentation process enhanced the nutritional value of TB. *Poria cocos* solid-state fermentation can improve the nutritional value of TB by increasing the content of bioactive substances and their release amounts during human digestion. It should be noted that the in vitro digestion model employed here only reflects bioaccessibility (the release of compounds from the food matrix), not true bioavailability, which requires in vivo studies involving absorption, metabolism, and systemic distribution. Nevertheless, this model is widely accepted as an initial screening tool, and similar approaches have been used in related studies [22].

To study the antioxidant activity changes of fermented products during human digestion, this research simulated oral–gastric–intestinal digestion in vitro at different fermentation stages and measured DPPH radical scavenging ability, ABTS radical scavenging activity, and ferric reducing antioxidant power (FRAP) in three digestion phases. However, DPPH radical scavenging activity is highly sensitive to pH and matrix effects, and the varying environmental conditions during in vitro gastric and intestinal digestion may affect assay reliability independently of the true antioxidant capacity. For this reason, direct quantitative comparisons of absolute DPPH values between the original fermented products (Table 6) and different digestion phases should be avoided. Nevertheless, a consistent trend was observed throughout all three digestion stages: the fermented samples always showed higher DPPH radical scavenging activity than the unfermented samples. This indicates that fermentation with *Poria cocos* effectively improves antioxidant activity, and this positive effect remained stable across the entire in vitro digestion process. As shown in Table 9, during digestion, FTB had a high DPPH scavenging ability in oral digestion, with FTB-5 reaching 98.65%, which was 13.32% higher than NFTB (85.33%). In gastric digestion, NFTB’s DPPH scavenging ability rose from 85.33% to 97.35%. FTB’s DPPH scavenging ability showed no obvious change during GI digestion. DPPH and ABTS assays rely on different reaction mechanisms. The increase in DPPH radical scavenging activity is primarily attributed to the release of aglycones from their glycosides via microbial glycosidases secreted during the fermentation of *Poria cocos*. These free phenolic compounds possess a strong hydrogen-donating capacity. Conversely, the decrease in ABTS activity is likely influenced by the acidic microenvironment generated by the fermentation process. According to Table 8, the fermented product contains high levels of organic acids (e.g., 2-Methyl propanoic acid) with significant ROAV values. The acidic pH may have suppressed the antioxidant efficiency of certain components or caused intermolecular complexation, leading to a lower ABTS value. The volatile compounds identified in Table 8 may also exert a quenching effect on the ABTS assay system, which is more susceptible to matrix interference compared to the DPPH system. In conclusion, while the absolute values differ, the overall antioxidant potential of the Tartary buckwheat was enhanced by fermentation, as evidenced by the increased DPPH activity. Through the three different antioxidant assays, the result showed that TB by *Poria cocos* SSF had higher antioxidant activities during in vitro oral–GI digestion. Moreover, the high antioxidant activity of the samples was well reflected during the oral phase. Previous studies have shown that the antioxidant activity of TB is closely related to its phenolic compounds [4]. A similar result was obtained in this study, as the antioxidant capacity of the digestive fluid increased with the release of phenolic compounds. In addition to phenols and flavonoids, triterpenes and polysaccharides also possess antioxidant activity, and their increasing release affected the antioxidant capacity of the digestive fluid. This finding was consistent with Zhang’s view which suggested that the enhanced antioxidant activity of *Ganoderma lucidum*-fermented TB was primarily due to the increased content of active substances such as triterpenes, total phenols, and flavonoids [61].

SEM is a novel electro-optical instrument widely used in biology, medicine, metallurgy, and other fields. It possesses distinct advantages over conventional microscopes, such as high resolution, wide adjustable magnification, a large depth of field, and simple sample preparation. In this study, SEM was used to observe the morphology of TB powder before and after fermentation. The SEM images are shown in Figure 2G,H. SEM images revealed that FTB-5 exhibited an intact shape, compact texture, relatively smooth surface, and almost no internal pore structure. Before fermentation, after inoculation with *Poria cocos*, the mycelia secreted extracellular enzymes including cellulase and xylanase, which degraded rigid components such as cellulose and hemicellulose in the cell wall of buckwheat bran. As a result, the compact structure of the bran particles was destroyed and numerous voids were formed [62], leading to increased surface porosity, obvious corrosion, and a three-dimensional porous structure. After full colonization by *Poria cocos* and fermentation for 5 days, the mycelia entered a stable growth phase and continued to proliferate and interweave, forming a dense mycelial film on the surface of the bran particles. This film covered the original pores and transformed the surface from porous to smooth. These observations were consistent with the study by Yang et al., who reported that mulberry bark fermented for 28 days produced smooth-surfaced fibers [63]. We hypothesized that the voids may have been filled by degraded products, such as small-molecule polysaccharides, reducing sugars, and other soluble substances, which further reduced structural porosity and contributed to a smoother surface. Autolysis of mycelia may also play a supporting role. This interpretation remains speculative and should be validated in future studies. In conclusion, SEM analysis demonstrated that enzymatic hydrolysis and fermentation effectively modified the internal structure of buckwheat bran.

### 3.5. The Effect of Fermentation on Application Quality in TB CSB

**Figure 3:**
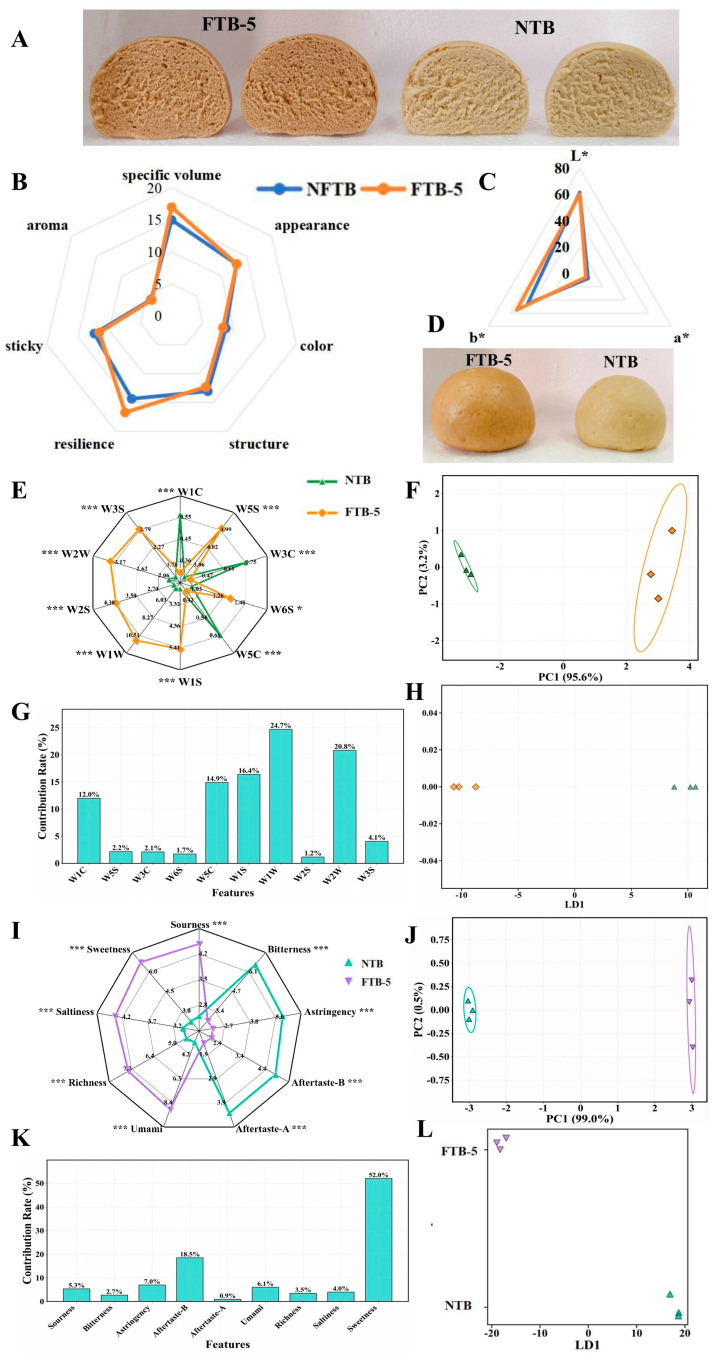
Cross-Section of TB CSB (**A**). The radar chart of sensory evaluation (**B**). The color of TB CSB (**C**). The appearance of TB CSB (**D**). Electronic nose analysis of aroma characteristics of TB CSB: Radar chart (**E**), Score plot (**F**), Contribution rate plot (**G**), and LDA discriminant analysis plot (**H**). Electronic tongue analysis of taste characteristics of TB CSB: Radar chart (**I**), Score plot (**J**), Contribution rate plot (**K**), and LDA discriminant analysis plot (**L**). Note: *, **, and *** indicate significant differences at *p* < 0.05, *p* < 0.01, and *p* < 0.001, respectively.

CSB has remained a predominant dietary staple among indigenous populations, owing to its profound socio-cultural significance, nutritional superiority, and distinctive organoleptic properties [64]. Consumer preference and the regional prevalence of CSB are predominantly determined by its sensory appeal, particularly its flavor complexity and textural attributes, which collectively define product acceptability and culinary identity [65,66]. As shown in Figure 3A, among the two types of CSB, FTB CSB had a larger specific volume, a sponge-like soft structure, and good elasticity and toughness. It can be seen from Figure 3B that FTB-5 performed better than NFTB in terms of specific volume, aroma, elasticity, appearance, and color. Although FTB-5 was also slightly better than NFTB in terms of stickiness and structure, the gap was relatively small. Their color was brown, their odor was slightly worse, and they were somewhat sticky to the teeth (Figure 3C,D). Combined with Figure 3A, the performance advantages of FTB-5 in Figure 3B were also reflected to some extent visually, and FTB-5 looked fluffier, which was consistent with its high score in specific volume. Due to the large proportion of the specific volume score, FTB-5 CSB obtained a higher comprehensive sensory score. The color of cereals was associated with factors such as bioactivity and the sensory quality of final products [67]. The color of the TB CSB is shown in Figure 3C. Fermentation decreased the a* value of TB, while the b* value increased, indicating that as the fermentation time increased, the color of TB changed from red-blue to green-yellow. Fermentation reduced the L* value of TB, and the observed darkening could be attributed to the oxidation of phenolics during fermentation [11]. Compared with the unfermented samples, the FTB-5 CSB had a relatively large ΔE value of 9.94, so the obvious color difference between the two samples can be directly seen by the human eye. As shown in Figure 3B, the specific volume of the FTB CSB increased significantly, which may be affected by soluble dietary fiber. Some studies have shown that an appropriate amount of soluble dietary fiber can increase the specific volume of CSB [13,68]. In summary, FTB with *Poria cocos* can be used to prepare CSB with good sensory qualities.

Based on the electronic nose detection results and multivariate statistical analysis, the mechanism by which fermentation affects the flavor formation of the samples can be clarified. During fermentation, microbial metabolism may activate a variety of biochemical pathways, including protein hydrolysis, lipid oxidation, Maillard reaction, and sulfur-containing amino acid degradation, potentially generating large amounts of aromatic compounds, sulfides, alcohols, and alkanes, which could endow the fermented samples with rich characteristic flavors (Figure 3E). Compared with buckwheat steamed bread, fermented buckwheat steamed bread showed a significantly increased response value of the W1W sensor (11.11 vs. 4.47), indicating that sulfur-containing amino acids (e.g., methionine and cysteine) were degraded by microorganisms during fermentation, producing abundant sulfide flavor compounds. This is consistent with the observed slight decrease in methionine content (from 0.14 to 0.12, Table 5), which may be attributed to its microbial conversion into volatile sulfur compounds. The decreased characteristic values of the W1C sensor (0.31 vs. 0.56; a smaller value after reciprocal transformation represents a stronger response) and W5C sensor (0.36 vs. 0.66) suggested that aromatic compounds and alkanes increased significantly during fermentation, which was closely related to the volatile organic acids, aldehydes, and esters generated via microbial metabolism. The response value of the W2S sensor increased from 2.12 to 4.26, reflecting the accumulation of alcohols during fermentation, which were mainly derived from microbial carbohydrate metabolism and Strecker degradation of amino acids. The response value of the W6S sensor (hydrogen) changed from 0.93 to 1.32, and the characteristic value of the W3C sensor changed from 0.75 to 0.40, further reflecting the complex biochemical reactions during fermentation. These findings provide a scientific basis for the optimization of fermentation processes, allowing the target flavor profile to be tailored by regulating fermentation conditions. PCA is one of the most commonly used pattern recognition methods for electronic nose. Through data transformation and dimensionality reduction of sensor responses, followed by linear classification, PCA enables sample classification and difference analysis. The PCA results in Figure 3E show that the cumulative variance contribution rate of the first two principal components (PC1 and PC2) reached 98.8%, with PC1 accounting for 95.6% and PC2 for 3.2%, indicating that these two principal components can effectively characterize the differences in volatile components between the two samples. As observed from Figure 3F, the NTB CSB and FTB-5 CSB were completely separated in the two-dimensional space, and their 95% confidence ellipses were independent without any overlapping regions, demonstrating that fermentation exerted an essential effect on the volatile composition of the samples. FTB CSB showed a larger projection in the positive direction of PC1, suggesting that fermentation produced richer volatile components. The discriminant model established by Linear Discriminant Analysis (LDA) achieved an overall discriminant accuracy of 100.00% for the two samples. According to Figure 3G, sensors such as W1W, W5S, and W1S exhibited high weights in the discriminant function, indicating that their responses to sulfides, nitrogen oxides, and methane compounds are key indicators for distinguishing the two samples. Figure 3H presents the LDA discriminant analysis plot, in which LD1 clearly separated the two samples in the discriminant space, and the 95% confidence ellipses of each group were independent. These findings are fully consistent with our GC-IMS volatile profile. For example, the higher responses of W1S in FTB-5 correspond to the presence of fruity esters (geranyl formate). In contrast, the relatively higher responses of W1C/W5C in NTB are consistent with the presence of aromatic aldehydes (e.g., 2-furaldehyde), which contribute to the bitter and almond-like notes. The results further confirm that the influence of fermentation on sample flavor is highly distinguishable. This provides a reliable basis for the rapid identification and quality evaluation of fermented samples using electronic nose technology.

As shown in Figure 3I, significant differences (*p* < 0.05) were observed in the response values of the nine taste attributes between the NFB CSB and FTB-5 CSB. The response values of the NTB CSB were 6.61 for bitterness, 5.30 for astringency, 4.86 for aftertaste bitterness, and 4.30 for aftertaste astringency, showing strong bitter and astringent characteristics. The umami value was only 3.16 and the sweetness value was 2.27, indicating weak umami and sweet tastes. The FTB-5 CSB exhibited significantly different taste profiles. The bitterness, astringency, aftertaste bitterness, and aftertaste astringency decreased to 2.75, 2.15, 1.95, and 1.46, respectively, demonstrating that fermentation remarkably reduced the bitter and astringent tastes. The umami value increased to 8.96, which was 2.83 times that of the NTB CSB, suggesting that fermentation promoted the release of umami substances such as amino acids. The sweetness and richness reached 6.71 and 7.94, respectively, indicating that fermentation significantly improved the overall taste quality. The sourness increased from 2.52 to 4.48, and the saltiness from 3.03 to 4.30. The increase in sourness was closely related to the production of organic acids during fermentation, and the moderate accumulation of organic acids helped enhance the layering and coordination of the overall flavor [69]. The acidification process was accompanied by protein degradation, and a large amount of taste-active free amino acids were released under the combined action of organic acids and proteases. Furthermore, free amino acids could serve as precursors for taste peptides, and under the action of enzyme systems, they were converted into small peptides with various tastes, contributing to the overall taste characteristics of the product. [70]. To further clarify the differences in the overall taste characteristics of CSB samples, PCA was performed based on the electronic tongue sensor and pattern recognition system to classify and distinguish the samples. As shown in Figure 3J, the cumulative variance contribution rate of the first two principal components (PC1 and PC2) reached 99.54%, with PC1 accounting for 99.01% and PC2 for 0.53%, indicating that the two principal components could effectively represent the taste differences between the two samples. The score plot showed that the NTB CSB and FTB-5 CSB were completely separated in the two-dimensional space, and their 95% confidence ellipses were independent without overlapping, demonstrating that fermentation exerted an essential effect on the taste characteristics of the samples. The discriminant model established by LDA achieved an overall discriminant accuracy of 100.00% for the two samples. As shown in the feature contribution plot (Figure 3K), umami, bitterness, sweetness, and other taste attributes had high weights in the discriminant function, indicating that these taste dimensions were the key indicators for distinguishing the two samples. Figure 3L presents the LDA discriminant plot, in which LD1 clearly separated the two samples in the discriminant space, and the 95% confidence ellipses of each sample were independent. The PCA and LDA plots (Figure 3J,L) showed complete separation between NTB and FTB-5, indicating distinct taste and aroma profiles between the two samples. Electronic tongue analysis (Figure 3I,K) further revealed that the primary drivers of this separation were sweetness (52.0%) and aftertaste-B (18.5%), with secondary contributions from astringency (7.0%) and umami (6.1%). These taste differences were consistent with the volatile profiles obtained by GC-IMS. Specifically, FTB-5, which exhibited higher levels of sweetness and richness, and contained higher levels of fruity and sweet-tasting geranyl formate, whereas NTB, characterized by stronger bitterness and astringency, had higher contents of bitter/astringent aldehydes (e.g., benzaldehyde, heptaldehyde). These results confirm that the fermentation treatment significantly altered the taste-active components, leading to the distinct sensory profiles observed in the multivariate analysis. This further confirmed that the influence of fermentation on the taste characteristics of the samples was highly distinguishable. These results provide a reliable basis for the rapid identification and quality evaluation of fermented products using electronic tongue technology.

## 4. Conclusions

This study demonstrates that 5-day SSF of TB with *Poria cocos* is optimal, significantly enhances the bioaccessibility of bioactive compounds, and produces better-quality steamed bread. Specifically, FTB-5 increased the amount of protein, polysaccharides, reducing sugars, triterpenes, total phenols, and flavonoids; improved in vitro bioaccessibility; optimized aroma (decreased alcohols/furans, increased furanones/hydrocarbons); and yielded CSB with a superior texture, color, and sensory score. These findings support the application of *Poria cocos*-fermented TB as a functional food ingredient.

## Figures and Tables

**Table 1:** Dynamic changes in various nutrients and antioxidant activities during SSF.

Groups	Protein (mg/g)	Polysaccharide (mg/g)	Reducing Sugar (mg/g)	Flavonoid (mg/g)	Triterpene (mg/g)	Total Phenolic (mg/g)	DPPH Free Radical Scavenging Activity (%)
NFTB	103.00 ± 2.45 ^b^	219.92 ± 2.40 ^f^	12.83 ± 0.24 ^f^	22.17 ± 1.16 ^c^	3.79 ± 0.40 ^d^	11.25 ± 0.20 ^d^	90.96 ± 1.31 ^b^
FTB-5	119.00 ± 0.82 ^a^	567.39 ± 3.81 ^b^	515.30 ± 30.23 ^b^	23.40 ± 0.15 ^c^	11.19 ± 0.57 ^bc^	16.80 ± 0.03 ^c^	95.83 ± 1.14 ^a^
FTB-10	105.00 ± 1.14 ^b^	508.54 ± 8.04 ^c^	407.63 ± 16.21 ^c^	23.03 ± 0.27 ^c^	9.92 ± 0.30 ^c^	17.39 ± 0.21 ^b^	91.09 ± 1.45 ^b^
FTB-15	102.50 ± 0.57 ^b^	669.29 ± 6.45 ^a^	549.30 ± 8.98 ^a^	19.20 ± 0.42 ^d^	9.67 ± 0.20 ^c^	18.83 ± 0.05 ^a^	85.71 ± 1.52 ^c^
FTB-20	103.50 ± 0.41 ^b^	293.03 ± 3.66 ^e^	182.77 ± 0.42 ^e^	31.73 ± 1.18 ^a^	14.02 ± 0.94 ^a^	16.74 ± 0.14 ^c^	86.03 ± 1.21 ^c^
FTB-25	98.20 ± 0.45 ^c^	369.25 ± 9.56 ^d^	199.22 ± 0.41 ^d^	27.29 ± 0.20 ^b^	12.72 ± 0.92 ^ab^	16.71 ± 0.06 ^c^	89.29 ± 2.00 ^b^

Note: Different superscript letters of the same column data indicate significant differences (*p* < 0.05), while the same superscript letters indicate no significant differences (*p* > 0.05).

**Table 2:** PCA explains total variables for nutrients and antioxidant indicators of TB for different components.

Principal Component	Factor Extraction Result		
	Eigenvalue	Variance Contribution Rate (%)	Cumulative Contribution Rate (%)
1	3.214	45.909	45.909
2	2.225	31.787	77.696
3	1.374	19.634	97.331
4	0.176	2.518	99.849
5	0.011	0.151	100
6	0.000	0.000	100
7	0.000	0.000	100

**Table 3:** Principal component loading matrix of quality indexes of TB for different contents.

Indexes	PC1 (45.603%)	PC2 (31.106%)	PC3 (19.375%)
Protein content	0.277	0.019	0.92
Polysaccharide content	0.974	−0.178	0.133
Reducing sugar content	0.967	−0.047	0.245
Flavonoid content	−0.415	0.902	−0.097
Triterpene content	0.392	0.911	−0.071
Total phenolic content	0.894	0.405	−0.169
DPPH free radical scavenging activity	−0.085	−0.167	0.938

**Table 4:** Composites scores and rankings of TB for different groups.

Groups	Y1	Y2	Y3	Y	Ranking
NFTB	−1.53932	−1.29417	0.05766	−1.14	6
FTB-5	0.53857	0.19176	1.86455	0.69	1
FTB-10	0.41201	−0.19905	0.09663	0.15	3
FTB-15	1.36091	−0.81741	−0.99074	0.18	2
FTB-20	−0.48726	1.46918	−0.49492	0.15	4
FTB-25	−0.28491	0.64969	−0.53318	−0.03	5

**Table 5:** Amino acid composition and content of TB before and after fermentation.

Groups	NFTB	FTB-5
Total content	9.31%	10.46%
Aspartic acid	1.01%	1.14%
Glutamic acid	1.68%	1.90%
Histidine	0.27%	0.33%
Serine	0.57%	0.48%
Arginine	0.88%	1.01%
Glycine	0.43%	0.71%
Threonine	0.38%	0.48%
Proline	0.44%	0.48%
Alanine	0.47%	0.58%
Valine	0.55%	0.62%
Methionine	0.14%	0.12%
Cystine	0.00%	0.00%
Isoleucine	0.49%	0.50%
Leucine	0.76%	0.84%
Phenylalanine	0.55%	0.56%
Lysine	0.49%	0.48%
Tyrosine	0.17%	0.23%

**Table 6:** Antioxidant activity, antinutritional factors (phytic acid and tannin), and α-Amylase inhibitory activity of TB before and after fermentation.

Groups	NTB	FTB
ABTS radical scavenging activity (%)	71.07 ± 0.13 ^a^	40.17 ± 0.30 ^b^
DPPH radical scavenging activity (%)	90.96 ± 1.31 ^b^	95.83 ± 1.14 ^a^
Fe^2+^ (μmol/g)	14.11 ± 0.03 ^b^	16.39 ± 0.04 ^a^
phytic acid (mg/g)	2.99 ± 0.07 ^a^	2.60 ± 0.01 ^b^
tannin (mg/g)	1.55 ± 0.05 ^a^	1.57 ± 0.02 ^a^
α-Amylase inhibitory activity (%)	53.94 ± 0.45 ^b^	71.87 ± 0.14 ^a^

Values with different lowercase letters indicate significant differences at *p* < 0.05.

**Table 7:** Volatile components of TB before and after fermentation.

	Aldehydes	CAS	RI	RT	DT	NFTB	FTB-5
1	benzaldehyde-M	C100527	1496.6	941.26	1.15417	1.32 ± 0.03%	0.71 ± 0.00%
2	benzaldehyde-D	C100527	1497.2	942.499	1.46879	0.09 ± 0.00%	0.04 ± 0.01%
3	3-Methyl-2-butenal	C107868	1211	434.242	1.08846	0.10 ± 0.01%	0.04 ± 0.01%
4	n-Pentanal	C110623	999.7	215.384	1.41847	0.70 ± 0.00%	0.19 ± 0.01%
5	Heptaldehyde-D	C111717	1194.1	409.987	1.68658	0.39 ± 0.02%	0.09 ± 0.00%
6	Heptaldehyde-M	C111717	1195.2	411.543	1.33078	1.01 ± 0.01%	0.41 ± 0.01%
7	Propanal	C123386	818.8	133.37	1.14205	1.41 ± 0.04%	1.80 ± 0.02%
8	Butanal-D	C123728	892.8	162.162	1.27756	0.26 ± 0.02%	0.07 ± 0.00%
9	Butanal-M	C123728	891.6	161.616	1.11607	0.31 ± 0.00%	0.00 ± 0.01%
10	Octanal-M	C124130	1301.4	586.317	1.40262	0.42 ± 0.01%	0.12 ± 0.01%
11	Octanal-D	C124130	1300.7	585.289	1.81211	0.17 ± 0.01%	0.07 ± 0.01%
12	nonanal-M	C124196	1400.2	745.036	1.47913	1.29 ± 0.02%	0.50 ± 0.01%
13	nonanal-D	C124196	1400.9	746.2	1.9372	0.17 ± 0.01%	0.04 ± 0.01%
14	(E)-2-Pentenal	C1576870	1144.6	344.552	1.10624	0.18 ± 0.01%	0.04 ± 0.00%
15	(E)-2-Heptenal-D	C18829555	1340	643.849	1.6623	0.36 ± 0.02%	0.02 ± 0.01%
16	(E)-2-Heptenal-M	C18829555	1340.9	645.187	1.25262	2.03 ± 0.02%	0.18 ± 0.04%
17	3-Methyl butanal-M	C590863	925.1	176.558	1.16011	0.12 ± 0.01%	0.09 ± 0.01%
18	3-Methyl butanal-D	C590863	927	177.47	1.39838	2.45 ± 0.05%	3.38 ± 0.06%
19	hexanal-D	C66251	1099.1	293.455	1.55723	5.79 ± 0.11%	1.03 ± 0.03%
20	hexanal-M	C66251	1097.7	292.025	1.26898	3.03 ± 0.06%	1.42 ± 0.05%
21	(E)-2-hexenal	C6728263	1230.4	463.703	1.17959	0.56 ± 0.02%	0.13 ± 0.03%
22	2-Methyl propanal	C78842	831.5	137.926	1.27869	0.64 ± 0.01%	0.73 ± 0.01%
23	2-furaldehyde-D	C98011	1460	861.308	1.33032	13.27 ± 0.29%	13.60 ± 0.25%
24	2-furaldehyde-M	C98011	1456.1	853.252	1.08431	4.51 ± 0.11%	4.07 ± 0.13%
	**Esters**						
25	geranyl formate	C105862	1692.9	1515.129	1.22775	0.21 ± 0.05%	0.51 ± 0.06%
26	Acetic acid propyl ester	C109604	967.5	197.499	1.47224	0.06 ± 0.00%	0.11 ± 0.00%
27	Ethyl caproate-D	C123660	1225	455.35	1.81521	1.38 ± 0.03%	1.26 ± 0.04%
28	Ethyl caproate-M	C123660	1226	456.869	1.34224	4.24 ± 0.04%	4.03 ± 0.07%
29	butyl acetate-D	C123864	1084.4	280.122	1.6155	0.90 ± 0.04%	0.45 ± 0.01%
30	butyl acetate-M	C123864	1085.1	280.775	1.2359	1.36 ± 0.04%	0.95 ± 0.01%
31	Butyl 2-propenoate-M	C141322	1185	397.255	1.68224	0.20 ± 0.00%	0.15 ± 0.00%
32	Butyl 2-propenoate-D	C141322	1185.7	398.19	1.26112	0.69 ± 0.01%	0.59 ± 0.01%
33	Acetic acid ethyl ester	C141786	900.4	165.442	1.33628	1.00 ± 0.01%	0.11 ± 0.00%
34	Acetic acid hexyl ester	C142927	1294.6	576.393	1.38398	0.16 ± 0.00%	0.18 ± 0.00%
35	Butyl propanoate-D	C590012	1149.9	350.981	1.71603	1.33 ± 0.02%	1.13 ± 0.05%
36	Butyl propanoate-M	C590012	1148.2	348.85	1.28187	1.72 ± 0.03%	2.24 ± 0.03%
	**Alcohols**						
37	1-hexanol	C111273	1368	689.139	1.32153	0.42 ± 0.01%	0.21 ± 0.02%
38	2-methyl-1-butanol	C137326	1215.9	441.53	1.23525	0.14 ± 0.00%	0.28 ± 0.01%
39	1-Penten-3-ol	C616251	1169.9	376.591	0.94368	0.20 ± 0.01%	0.06 ± 0.00%
40	Ethanol	C64175	945.4	186.321	1.13193	4.82 ± 0.27%	1.55 ± 0.02%
41	Propanol	C71238	1049.7	251.498	1.11091	0.09 ± 0.00%	0.06 ± 0.00%
42	Butanol-M	C71363	1151.6	353.111	1.17989	3.57 ± 0.05%	2.47 ± 0.014%
43	Butanol-D	C71363	1155.9	358.438	1.37803	2.14 ± 0.03%	0.95 ± 0.03%
44	1-Pentanol-M	C71410	1260.5	513.608	1.25302	1.24 ± 0.02%	0.26 ± 0.02%
45	1-Pentanol-D	C71410	1260.3	513.277	1.51233	0.17 ± 0.00%	0.02 ± 0.00%
46	2-Methyl-1-propanol	C78831	1103.8	298.384	1.17139	0.10 ± 0.01%	0.31 ± 0.01%
	**Ketones**						
47	4-Methyl-2-pentanone	C108101	1024.2	232.424	1.17939	0.62 ± 0.02%	0.33 ± 0.01%
48	Cyclohexanone	C108941	1292.8	572.886	1.1548	0.56 ± 0.04%	0.29 ± 0.03%
49	6-methyl-5-hepten-2-one	C110930	1354.9	667.486	1.1752	0.10 ± 0.00%	0.06 ± 0.00%
50	3-Hydroxy-2-butanone-M	C513860	1292.1	571.6	1.06713	2.39 ± 0.06%	4.97 ± 0.21%
51	3-Hydroxy-2-butanone-D	C513860	1294.4	576.06	1.3252	0.53 ± 0.00%	2.46 ± 0.11%
52	3-Penten-2-one	C625332	1140.8	339.948	1.07509	0.09 ± 0.00%	0.03 ± 0.00%
53	2-propanone	C67641	836	139.566	1.11043	3.74 ± 0.08%	2.94 ± 0.05%
54	3,5,5-Trimethyl-2-cyclohexen-1-one	C78591	1609.3	1237.068	1.25542	0.27 ± 0.03%	0.74 ± 0.01%
55	2-Butanone-D	C78933	917.7	173.154	1.24602	0.61 ± 0.04%	0.58 ± 0.02%
56	2-Butanone-M	C78933	919.1	173.79	1.06243	0.18 ± 0.01%	0.21 ± 0.00%
	**Carboxylic acids**						
57	Butanoic acid-D	C107926	1636.3	1320.738	1.36578	0.16 ± 0.06%	0.76 ± 0.11%
58	Butanoic acid-M	C107926	1635	1316.75	1.16946	0.19 ± 0.02%	1.59 ± 0.15%
59	Acetic acid-M	C64197	1473	888.93	1.05233	10.81 ± 0.43%	7.43 ± 037%
60	Acetic acid-D	C64197	1470.9	884.327	1.16057	3.25 ± 0.07%	1.28 ± 0.10%
61	2-Methyl propanoic acid-D	C79312	1581.1	1155.395	1.36294	0.08 ± 0.04%	1.51 ± 0.14%
62	2-Methyl propanoic acid-M	C79312	1581.9	1157.502	1.15574	0.89 ± 0.08%	8.31 ± 038%
	**Furanones**						
63	2(3H)-Furanone, 5-methyl-D	C591128	1428.3	797.641	1.38217	2.83 ± 0.04%	12.79 ± 0.28%
64	2(3H)-Furanone, 5-methyl-M	C591128	1423.6	788.431	1.12647	4.86 ± 0.09%	6.22 ± 0.13%
	**Ethers**						
65	Tetrahydrofuran-D	C109999	882.9	157.971	1.22674	0.06 ± 0.00%	0.01 ± 0.00%
66	Tetrahydrofuran-M	C109999	884.7	158.7	1.063	0.12 ± 0.01%	0.07 ± 0.00%
	**Pyrazines**						
67	2-ethyl-3,5-dimethylpyrazine	C13925070	1469.6	881.637	1.22185	0.63 ± 0.02%	0.35 ± 0.02%
	**Furans**						
68	2-Pentylfuran	C3777693	1238.9	477.332	1.24956	0.09 ± 0.01%	0.02 ± 0.00%
	**Thioethers**						
69	diethyl disulfide	C110816	1234.7	470.512	1.13791	0.09 ± 0.015%	0.06 ± 0.00%
	**Hydrocarbons**						
70	1-heptene	C592767	792.3	124.378	1.08772	0.15 ± 0.01%	0.27 ± 0.02%

Note: D—dimer, and M—monomer.

**Table 8:** Odor contribution of volatile components of TB before and after fermentation.

Key Compounds	ROAV			Sensory Description
	VIP Score	NFTB	FTB-5	
2(3H)-Furanone, 5-methyl-M	1.57453	0.170414201	1.427272727	Spicy, roasted sweet aroma
2(3H)-Furanone, 5-methyl-D	3.57775	0.170414201	1.427272727	Spicy, roasted sweet aroma
2-Methyl propanoic acid-M	3.0542	0.100591716	7.181818182	buttery, cheesy
2-Methyl propanoic acid-D	1.33267	0.017751479	3.409090909	buttery, cheesy
hexanal-M	1.21305	5.776275007	47.619047619	grassy, tallowy, fat
hexanal-D	2.21351	15.046491970	37.445887446	grassy, tallowy, fat
3-Hydroxy-2-butanone-M	1.88407	0.059171598	1.072727273	creamy
3-Hydroxy-2-butanone-D	1.57423	0.020118343	1.131818182	creamy
Acetic acid-M	1.45351	0.073695535	0.510330579	sour
Acetic acid-D	1.37178	0.059440559	0.235537190	sour
2-furaldehyde-D	1.63358	4.337278107	38.409090909	almond oil flavor, burnt, flower, fruity
Ethanol	1.78596	0.000791675	0.003495298	pungent, ether
(E)-2-Heptenal-M	1.39531	100.000000000	100.000000000	fatty, fruity, green
Butanoic acid-M	1.32476	0.017339662	1.115551116	cheesy
3-Methyl butanal-D	1.25367	0.009397842	0.097058824	flower, fruit
Butanol-D	1.04904	0.222485207	1.427272727	medicinal, fruit
1-Pentanol-M	1.00523	0.088421194	0.002066116	fruity

Note: Substances whose threshold values could not be found are not displayed.

**Table 9:** Changes of antioxidant capacity of TB in vitro digestion simulation for different groups.

Groups		Oral Phase	Gastric Phase	Intestinal Phase
DPPH scavenging activity (%)	NFTB	85.33 ± 3.0	97.35 ± 1.8	98.73 ± 1.0
	FTB-5	98.65 ± 1.1	99.14 ± 1.0	99.32 ± 0.3
ABTS scavenging activity (%)	NFTB	40.17 ± 0.30	47.64 ± 0.71	43.40 ± 0.34
	FTB-5	55.06 ± 0.07	61.63 ± 0.71	57.92 ± 0.07
Fe^2+^ (μmol/g)	NFTB	212.02 ± 1.88	322.28 ± 6.38	90.21 ± 1.64
	FTB-5	325.75 ± 5.67	330.09 ± 6.14	155.68 ± 1.66

## Data Availability

The original contributions presented in this study are included in the article. Further inquiries can be directed to the corresponding author.
